# Whither hydrocarbons? *The rescaling of global oil and gas markets amidst COVID-19 and a contested transition*

**DOI:** 10.1177/0308518X221114879

**Published:** 2022-11

**Introduction   1642**

Gabe Eckhouse and Anna Zalik

**COVID-19, global oil market volatility, and the renewable energy transition   1648**

Gabe Eckhouse

**Trading houses & market volatility: Global commodity traders and the shifting landscapes of oil and gas   1653**

Michael Watts

**On oil capitals and empire: The advance of international arbitration under COVID-19   1657**

Angus Lyall and Gaby Valdivia

**The perpetual rescaling of oil and gas production and flows in continental North America   1661**

Anna Zalik

**Expropriating hydrocarbons in the 21st century   1664**

Matt Huber

**Notes to section contributions   1668**
