# Supplementary material for: Whither hydrocarbons? The rescaling of global oil and gas markets amidst COVID-19 and a contested transition
Source: Environ Plan A. 2022 Nov;54(8):1641–68. doi: 10.1177/0308518X221114879 (PMC9486678; doi:10.1177/0308518X221114879)
Supplement: Related texts

# Other: Introduction

**Authors:** Gabe Eckhouse, Anna Zalik

**Affiliations:** University of California Berkeley, USA; 7991 York University, Toronto, Canada

Oil and gas markets are at a historic juncture. The initial measures taken across the world in March and April 2020 to combat the COVID-19 pandemic led to the largest absolute and relative decline in global hydrocarbon use on record. At the time, the International Energy Agency (IEA) suggested this downturn was a unique opportunity to shift energy investments towards renewables through significant government intervention (IEA, 2020). However, in the absence of deep-seated changes, and following the end of COVID-19 elimination measures in most of the world, fossil fuel demand skyrocketed back towards pre-pandemic levels. Big oil is no longer divesting hydrocarbon assets but has conducted the largest share buy back in history. This shift from bear to bull market clearly diverges from the purported death knell of oil and gas in 2020 (see Eckhouse, this section; Kuzemko et al., 2020). European tensions over gas sourcing, followed by the war in Ukraine, have brought about the greatest commodities boom since the 2008 financial crisis and the highest daily price rallies since the 1970s. Under the threat of a new round of ‘resource wars’, a range of spatially divergent assets are once again primed to be highly valued. For the hydrocarbon sector, the outcome is positive: counteracting demands for decarbonization, the oil industry significantly benefits from the higher oil and gas prices arising from war. Violence or the threat thereof in strategic oil and gas exporters prompts price increases globally; the beneficiaries include hydrocarbon producers far afield from the site of conflict. The general inflation in prices, exacerbated by the war in Ukraine, leads to windfall profits for fossil fuel producers – as witnessed in the Middle East throughout the 1990s and after 9–11 – as well as the states that house them, including in continental North America.

This ‘Exchanges’ section of Environment and Planning brings together six critical geographers of hydrocarbons in an analysis of this conjuncture. Through five interventions on five different aspects of the global industry, the authors shed collective light on how the conditions of crisis under COVID-19 have intersected with the political economy of hydrocarbons. Each of the contributions – in its own way – examines the oil and gas industry in the COVID-19 moment via a divergent scalar approach and/or unit of analysis. As we discuss further below, their contributions reveal that conjunctural analysis of this complex industry is well served by a set of four complementary approaches and units of analysis – ones that warrant ongoing, careful attention from critical geographers: First, the container of the *state* remains a significant scale of analysis, but its exploration requires constant vigilance to avoid methodological nationalism.

That is, the state must be de-reified and a consideration of its mutual constitution with other spatial units is essential to nuanced analyses of state energy politics and spatio-economic planning. Second, and concurrently, the increased attention to supply chain disruptions brought about by the COVID-19 crisis points to the need for *regional* approaches – that is, to analyses of spatially and terrestrially connected, frequently continental, markets and trade blocks. Third, the essential connection of oil and gas production to global markets highlights the importance of understanding concealed *mid-stream actors* in transnational hydrocarbons and their governance – trading firms, juridical structures and influential energy institutions, essential conduits in the oil and gas trade that shape trading options and future investments. Fourth, and finally, *global financial capital* remains a powerful force in energy markets – in its influence on prices and investment and as it shapes the future of energy, but also markedly unstable and dysfunctional.

These varied approaches and units of analysis underline the enormity and complexity of global oil and gas production circuits and trade (Bridge and Bradshaw, 2017). In 2020, oil and gas accounted for 57 percent of the world's primary energy use, a number which does not include non-energetic refined products and petrochemicals, including plastics. Global oil and gas markets were valued at $5.8 trillion in 2021 – accounting for more than 6 percent of the world's GDP (Research and Markets, 2021). Consequently, these brief contributions can only begin to highlight features of this inflexion point in a gargantuan industry. While empirically and methodologically distinct, these five different interventions collectively depict elements of the depth and heterogeneity of this multi-tendrilled, critical industry – one which defies simple summary.

The first two interventions, by Gabe Eckhouse and Michael Watts, provide complementary transnational points of departure. In a piece on *Covid, global oil market volatility, and the renewable energy transition,* Eckhouse explores how COVID-19 has exacerbated historic tendencies towards dysfunctional volatility in oil markets. Agents in the oil and gas industry frantically try to rescale to match the volatility that has destabilized capital mobilization in *both* renewable and fossil energies. Offering an introductory guide to the understudied world of global commodity trading houses, Watts’ *Global commodity traders and the shifting landscape of oil and gas* reveals these hidden midstream actors’ central position in capitalizing on, and leveraging, this recent extreme volatility in oil and gas markets.

The subsequent two pieces, by Angus Lyall & Gabriela Valdivia and Anna Zalik, offer analyses from the Americas that reveal the material and juridical infrastructures influencing regional and national oil and gas industry rescaling. In *On Oil Capitals and Empire: The Advance of International Arbitration Under COVID-19*, Lyall & Valdivia explore how disillusionment with national institutions in Ecuador has enabled the current neoliberal regime to move to rescale dispute resolution with transnational oil companies from local to international courts, bypassing national jurisdiction and reducing state capacities to (re)regulate oil production into the future. In *The perpetual rescaling of hydrocarbon production and flows in continental North America,* Zalik examines current features of North American continental import/export flows and reserve development as part of national energy sovereignties. Even under a renewed continental trade regime (NAFTA 2.0 – Canada Mexico United States Agreement – CUSMA/USMCA/TMEC) negotiated pre Covid that lifts some elements of corporate judicial power over hydrocarbon regulation, this has largely benefited Canadian and the US interests. The current Mexican government has sought, with limited success, to reinvigorate nation-state control over energy and hydrocarbons, and remains subjects under the new agreement to investor state arbitration in the energy sector even as this constraint has been removed for its Northern neighbors.

Finally, offering a normative reflection on the features revealed in the first two interventions on global markets, and echoing the constraints put on national and territorial sovereignties arising from foreign and private investment in the Americas, Matthew Huber offers a solution to the destabilizing influence of speculative capital for prospects for a non-hydrocarbon, just energy system. In *Expropriating Hydrocarbons in the 20^th^ century: Lessons from History,* Huber offers a scalar solution to this crisis point in fossil fuel transition informed by the history of oil expropriations in 19^th^ and 20^th^ century. His piece underscores the lessons from the previous four: the extreme market volatility bound up with COVID, and related supply shocks, require an energy system under the rule of non-market, social mechanisms.

## Rescaling and the Region

As an important basis for the discussion further below on the collective insights offered by the five interventions, we offer here a brief discussion of the concepts of rescaling and the region. These are concepts crucial to sub-disciplines of economic and resource geography, and which we argue warrant greater consideration in the conceptualization of oil and gas production, markets and futures in the present conjuncture.

Scale is both a central and evasive concept in human geography. Distinguished from its more common use as a ‘level’ or relative ‘size’, scale denotes, fundamentally, a relationship (Sayre and Di Vittorio, 2009). *Rescaling*, in this sense, is not about the sheer growth or decline of a level of social analysis, nor, for that matter, the absence or presence of said levels. Rather, rescaling is a process of rearticulation between levels, themselves constitutive of deeper social processes (Brenner, 1998: 299).

The oil and gas industry, pursuing the accumulation of capital as its *raison d’être*, necessarily structures itself on many levels. The industry's own primary triad of distinction is between upstream, midstream, and downstream, referring, respectively, to the extraction, transportation, and refinement of oil and gas. However, there are several other, by no means mutually exclusive, delineations with scalar implications. As we discuss below, oil and gas production necessarily operates on – and is therefore beholden, and in relationship to – global, continental, national, regional, as well as more local processes. The danger of understanding these processes as levels in too distinct a manner is revealed in the analyses provided by contributors to this section. For example, all the contributions confront what could be described as a *global* phenomenon of exceptional price volatility. While Eckhouse's intervention interrogates the dominant, dysfunctional relationship of global capital accumulation that lurks behind this price instability, Watts’ piece on commodity trading shows how *midstream* actors, beholden to their own variant of that logic, exploit and co-constitute the volatility in markets that must be conceived of as both regional and global. Meanwhile the contributions of Zalik and Lyall & Valdivia examine how this volatility has become a constitutive part of complex processes rearticulating relations between private capital, the state, and oppositional social actors on intertwined national, regional, and continental levels. While volatility provides just one example, together the pieces offer insight on rescaling processes underway in oil and gas production at this peculiar juncture on multiple co-constitutive levels – a spatial rearticulation of the relationships between capital accumulation, governance, geopolitics and social resistance. This rescaling can be viewed in the context of efforts to understand the instability of ‘carbonscapes’ at the present moment (Haarstad and Wanvik, 2017) and the need to champion non-market solutions (Huber, this section).

The ‘region’ is also crucial to understanding how rescaling processes in oil and gas production and markets are spatially mediated, particularly under the global supply chain disruptions of the Covid era and their association with the ‘end of globalization’. Shaping our understanding of the current moment, in the late 20^th^ century the ‘new regionalism’ crucially explained industrial and urban agglomeration and technological change (see, for instance, Walker and Storper, 1989).

Animated by the analysis of industrial growth, often in manufacturing, and sub/intranational regions (Massey, 1978) and their relations with broader national and global value chains, this work focused primarily on dynamics at the sub state scale. In parallel, classic essays in geography theorizing regions (Entrikin, 2017) ^1^ centre primarily on intra-national regions and less on continental terrestrial dynamics, although regional trade blocs – notably Europe – have received some attention. Nevertheless, the ‘resource frontiers’ within which oil and gas production are typically sited have largely remained external to this literature.

The critique of state-centric work across the social sciences, including examinations of territory beyond both the state (Agnew, 1994) and, more recently, the terrestrial (Peters, Steinberg and Stratform) offer important insights to studying oil and gas markets as transnational phenomena. Indeed, the push to think beyond both the state and the terrestrial is crucial to conceptualizing oil, gas, and other resources frontiers, from the global ocean (Havice and Zalik, 2018) to space exploration. That said, geographies and political economies of oil and gas in the COVID and ‘post globalization’ moment also demand attention to the perhaps more rudimentary, terrestrially connected, supranational market region that is the continent – whose role in energy security is underlined by the current war in Ukraine. Contributors to the section reveal how ‘regions’ under both transnational and intercontinental relations are mediated via terrestrial/continental proximity (Zalik this section) via market intermediaries (Watts this section), through global energy institutions (Eckhouse this section), transnational juridical structures (Lyall & Valdivia) and the actions of states operating to restrict the control of neoliberal market dynamics (Huber).

Alongside crucial studies on regionally connected sites in a broader global geography of oil (Labban, 2008) and national scale analyses (Appel, 2019 Huber, 2013; Kama, 2014; Sawyer, 2004; Valdivia, 2008), the present conjuncture – what some call ‘the end of globalization’ – reveals the weight of supra national regions and the forces that mediate them in the oil and gas market. Further theorization of continental dynamics – including the role of the physical reserve and infrastructure in shaping the *supra national energy region*, is warranted.

Empirically and theoretically, such work is particularly crucial at this pandemic and war-induced moment marked by supply chain constraints, constraints shaped by human geographies and geopolitical dynamics.

## Parallels and methodological insights: on the state, the region, midstream facilitators, and global finance in Covid era oil and gas markets

As summarized above – and to which we turn in more detail here, these pieces reveal that multiscalar analysis is well undertaken through a series of complementary approaches and units of analysis that offer insight on scalar variation in this complex, yet crucial market in the global economy.

First, these pieces, at this historic pandemic-induced conjuncture, point at the ongoing role of the container of *the state* in the global oil and gas market and in contentious extractive politics, notably as a key regulatory force in energy transitions. Indeed, various proponents of transition from fossil fuel stress the nation-state's role as essential to facilitating transformative restructuring of energetic systems (TUED, OilChange, ICA, IEN09). The position of the state in hydrocarbon politics, particularly as producer and exporter, is clearly central to the analysis in Huber, Lyall & Valdivia, and Zalik's contributions, with Huber in particular emphasizing the state's active ownership role and the historic figure of resource nationalization as essential to just transition. These analyses, equally, indicate that that the state must be examined via exploration of the historical and relational constitution of its social-territorial, including colonial, structures. Otherwise, the state can be incorrectly typologized as an entity separate from the global oil and gas sector in which it is embedded and that has shaped it. Thus, in analyzing the state, we must at once de-reify both the national and transnational legislative forms that give it form, and clearly – although less the focus in this exchanges section – the socio-cultural and discursive factors that make the state a meaningful entity to its residents (see Huber, 2013). This allows geographers to avoid the error of state reification common to much resource curse theory. Also crucial to a nuanced attention to the state, the role of our fourth methodological unit, global finance, clearly shapes the extent to which specific states can reshape sovereign control over hydrocarbons (as per the example of Mexico remaining subject to ISDS in the energy sector under TMEC – see Zalik). Sovereignty movements around energy (on the part of Indigenous peoples or states confronting imperial capital) influence a given state's ‘attractiveness’ to global capital, and thus its ability to access transnational financial investment; the threat of financial withdrawal or devaluation acts as a significant constraint on state resource autonomy.

Second, under Covid supply constraints in particular, *the supra-national region* – and notably the terrestrially connected continent – has been revealed as essential to protecting energy ‘security’ and hydrocarbon access. Importantly, following from the previous point on the state, international law and ‘private international law’ (for disputes between states and transnational companies) only materialize through state actions, whether through Bilateral Investment Treaties (BITs) or regional trade agreements, such as TMEC/CUSMA/USMCA. The international and national are co-constituted in these relationships. Over the last two years, the position of continental and subcontinental regions in energy flows has been accentuated both via restrictions in global trade arising from pandemic restrictions and supply chain blockages, and the current war in Ukraine. Notably Zalik and Lyall & Valdivia's interventions demonstrate the role of these regional containers for their impact on security of supply, and for continentally inflected autonomy (e.g. Ecuador's constitution only permits international investment arbitration *within* Latin America). Even as investment in LNG infrastructure, which was less attractive under pandemic lockdowns due to shipping constraints, has rebounded with the war in Ukraine, that same war had underlined how control over oil remains an essential marker of power in regional and global geopolitics. Indeed, as Watts’ analysis points out, global oil traders – and we would note their workers, subject to race and class exploitation – are central to making national oil companies viable and to spatially linking regional supply hubs. Alongside controversial pipeline projects and companies, these traders are major actors in making regions and in shaping inter-regional flows. Thus, the section encourages attention to continental trade blocs and their internal politics as empirical units of analysis, ones that shape oil and gas production and intra-regional market flows.

Third, the collection highlights the importance of transnational *midstream energy facilitators* that play an increasingly important role in the global oil and gas value chain. These often-hidden institutions and networks exert influence through their immediate, direct sway over energy markets, but also through their impact on the perceived future of oil and gas. Watts’ intervention, for example, explores the understudied world of trading houses. These shadowy players loom ever larger against today's backdrop of intensifying global volatility, geopolitical strife, and COVID-19 related disruption. The “intricate” and “opaque” commodity trading network has blossomed in an era of financialization and volatility. Eckhouse's contribution touches on another transnational actor – the International Energy Agency (IEA) – which, like the commodity trading houses, deals closely with the perceived future of the oil and gas industry.

While playing quite different roles – commodity trading houses and consequential global energy institutions engage in an incessant prediction and *preforming* of a global oil and gas future (see Christophers, 2014). A third entity that serves as a facilitator of hydrocarbon production and markets are international arbitration courts. Lyall & Valdivia reveal how the rising authority of international arbitration courts has been tied up with both global economic processes and broad social-political disillusionment with national institutions. If commodity trading houses and international energy prediction agencies both guide capital in its future pursuit of accumulation in energy markets, international arbitration courts insure investments in a tumultuous period. In turn, as Zalik highlights, regional market agreements also serve as less noted midstream facilitators, protecting financial and private capital from calls for nationalization (e.g. Mexico remains the only member of the NAFTA block subject to ISDS in the energy sector). Midstream actors, positioned between more global financial players and local extraction, therefore constitute important players in the rescaling of the industry taking place.

Finally, throughout all these contributions, the powerful force of *international financial capital* looms large, influencing and responding to fluctuations in a global oil market and changes in oil flows. As Hanieh (2021: 103) recently put it: ‘oil is as much a financial business as it is a physical commodity’. Each contribution deals in some way with the extreme price volatility and financial speculation that have shaped oil prices over the past two decades (Zalik, 2010). While each piece examines different phenomena, they offer reflections on how sections of the industry respond to global market tumult. As the piece by Lyall & Valdivia suggests, finance capital flows to where it can best control future investor-state disputes. In Zalik, finance influences the perpetual rescaling of North American hydrocarbon production. In Watts, a whole layer of financial players have made a livelihood off price volatility and speculations. Eckhouse's contribution deals with the question most directly, suggesting that the dysfunctional patterns of underinvestment in both renewable and fossil fuel energies will lead to a sustained and consequential period of extreme volatility for years to come. Collectively, we underscore the contradictory, intertwined relationship of oil and financial capital – not just abstractly – but as it constitutes spatially in relationship to consequential social processes.

A concern in these contributions is not just the question of international finance capital in terms of price, but finance capital in terms of its selection of targets of *investment,* themselves influenced by forces of labour and popular opposition. The commodity trading houses examined by Watts ultimately impact decisions over tens of billions of dollars of investments. In Lyall & Valdivia's contribution, we see that investment has become contingent upon access to arbitration tribunals for future disputes, and investment in Zalik's piece shapes or limits possibilities for resource renationalization in Mexico, given the threat of withdrawal of global finance capital. Eckhouse argues that the outmoded and irrational capitalist investment system is expressed in the inability of finance capital to adequately invest in either a renewable or fossil fuel future.

Arguably, Huber deals most directly with this threat on the part of capital and the need to counteract it through an ownership structure that removes the sector from control by private capital and finance. In confronting the intensifying climate catastrophe, and the complete inadequacy of tactics aimed to ‘nudge capital in the right direction’, Huber suggests the need to place state and collective expropriation of industry front-and-centre in political demands concerning the future of energy. We therefore document how this rescaling of oil and gas has direct consequences for the allotment of capital on regional, national, and global levels, and the impact this may have on the future of different scales of oil production.

Taken together the pieces deepen our collective understanding of the current volatility and uncertainty in global hydrocarbon markets. Exacerbated by COVID-19, but in no way beginning under the pandemic, hydrocarbon market instability has affected many different aspects of global social life. Collectively the section demonstrates the importance of complementary national, regional, and transnational scales and units of analysis. Through such trans-scalar examination, we can better understand and address the at once economic and socio-ecological crises facing industrial firms and capital blocs that shape the global hydrocarbon market. And we can begin to address the crucial question – *Given their inordinate influence on labour/ecology, how should the massive capital blocs in the political economy of this industry, from international oil companies – private and parastatal – to the servicing and infrastructural firms and financial arrangements around them, be controlled, and by whom?*

## References

[bibr1-0308518X221114879] AgnewJ (1994) The territorial trap: The geographical assumptions of international relations theory. Review of International Political Economy1(1): 53–80.

[bibr2-0308518X221114879] AppelH (2019) The Licit Life of Capitalism: US Oil in Equatorial Guinea. Durham: Duke University Press.

[bibr3-0308518X221114879] BrennerN (1998) Between fixity and motion: Accumulation, territorial organization and the historical geography of spatial scales. Environment and Planning D: Society and Space 16: 459–481.

[bibr4-0308518X221114879] BridgeGBradshawM (2017) Making a global gas market: Territoriality and production networks in liquefied natural gas. Economic Geography93(3): 215–240.

[bibr6a-0308518X221114879] Christophers B (2014) From Marx to market and back again: Performing the economy. *Geoforum; Journal of Physical, Human, and Regional Geosciences* 57: 12–20.

[bibr6-0308518X221114879] EntrikinJN (2017) Regions: Critical Essays in Human Geography. London: Routledge.

[bibr7-0308518X221114879] HaarstadHWanvikTI (2017) Carbonscapes and beyond: Conceptualizing the instability of oil landscapes. Progress in Human Geography41(4): 432–450.

[bibr8-0308518X221114879] HaniehA (2021) The commodities fetish? Financialisation and finance capital in the US oil industry. Historical Materialism29(4): 70–113.

[bibr9-0308518X221114879] HaviceEZalikA (2018) Ocean frontiers: Epistemologies, jurisdictions, commodifications. International Social Science Journal68(229–230): 219–235.

[bibr6b-0308518X221114879] Huber M (2013) *Lifeblood: Oil, Freedom, and the Forces of Capital*. Minneapolis: University of Minnesota Press.

[bibr10-0308518X221114879] International Energy Agency (IEA) (2020) Sustainable Recovery, Special Report, June 2020.

[bibr11-0308518X221114879] KamaK (2014) On the borders of the market: EU emissions trading, energy security, and the technopolitics of ‘carbon leakage’. Geopolitics51: 202–212.

[bibr12-0308518X221114879] KuzemkoCBradshawMBridgeG, et al. (2020) COVID-19 and the politics of sustainable energy transitions. Energy Research & Social Science68: 101685.3283970410.1016/j.erss.2020.101685PMC7330551

[bibr14-0308518X221114879] LabbanM (2008) Space, Oil and Capital. London: Routledge.

[bibr15-0308518X221114879] MasseyD (1978) Regionalism: Some current issues. Capital & Class2(3): 106–125.

[bibr17-0308518X221114879] Research and Markets (2021) Oil and Gas Global Market Report 2021: Covid-19 Impact and Recovery to 2030. https://www.researchandmarkets.com/

[bibr18-0308518X221114879] SawyerS (2004) Crude Chronicles: Indigenous Politics, Multinational Oil and Neoliberalism in Ecuador. Durham: Duke University Press.

[bibr19-0308518X221114879] SayreNFDi VittorioAV (2009) Scale. International Encyclopedia of Human Geography, Pages 19–28. 10.1016/B978-008044910-4.00318-7.

[bibr20-0308518X221114879] ValdiviaG (2008) Governing relations between people and things: Citizenship, territory, and the political economy of petroleum in Ecuador. Political Geography27(4): 456–477.

[bibr21-0308518X221114879] WalkerRStorperM (1989) The Capitalist Imperative: Territory, Technology and Industrial Growth (Vol. 2). Oxford: Basil Blackwell.

[bibr6c-0308518X221114879] Zalik A (2010) Oil ‘Futures’. *Geoforum* 41(4): 553–564.

# Other: COVID-19, global oil market volatility, and the renewable energy transition

**Authors:** Gabe Eckhouse

**Affiliations:** University of California Berkeley, USA

Amid global lockdowns at the onset of the COVID-19 crisis, some suggested the pandemic would accelerate the death of oil. ‘Oil Is Dying’, wrote *Forbes* (2020) in a headline. *Jacobin* (2020) stated the same month, in an article, *The Oil Industry is Dying Right Now, Don't Resuscitate It*, that ‘Significantly contracting oil output is now inevitable’. Carbon Tracker, a UK non-profit which ‘align[s] capital market actions with climate reality’, gave a fuller expression to this sentiment. They asserted in a June 2020 report that demand for oil had reached its peak in 2019 (see Figure 1). ‘The fossil fuel system is being disrupted by the forces of cheaper renewable technologies and more aggressive government policies’, they wrote. ‘In one sector after another, these are driving peak demand, which leads to lower prices, less profit, and stranded assets. The Covid-19 crisis is now accelerating this’.

**Figure 1.** Peak demand graphic from Carbon Tracker (2020), ‘Decline and Fall: The Size & Vulnerability of the Fossil Fuel System’.

This brief contribution complicates the notion that the COVID-19 pandemic accelerated a process of peak oil demand. I warn that oil is not so much dying as it is stuck in dysfunctional limbo. The anarchy of capitalist energy markets has reached new heights during the renewable energy transition – to the point that *neither* a renewable nor hydrocarbon-based future is being adequately invested in.

The initial lockdowns in Spring 2020 caused global oil consumption to drop from 96.6 million barrels per day (mb/d) in 2019 to 87.9 mb/d in 2020. This was the largest one-year fall in history. The downturn, however, was short-lived. With trillions of dollars of petroleum assets spatially embedded in cars, production, refinement, transportation, and more, demand grew globally as activity rebounded (Bridge et al., 2013). Oil demand increased an estimated 5.4 mb/d in 2021. A full recovery is expected by 2022. The rebound was so strong that carbon emissions grew by a record amount in 2021. The International Energy Agency (IEA) warned this was likely. In June 2020, they argued that the pandemic's downturn offered an opportunity for a renewable recovery, but an opportunity that would go unrealized without massive government investments.

As demand surged so did the price. Already, by November 2021, the Brent benchmark rose to $85 a barrel. *Bloomberg,* at the time, proclaimed the ‘Revenge of Fossil Fuels’ as high transportation costs dug into people's lives. Now, the escalating war in Ukraine has pushed the price even higher – above $100 for the first time since the 2014 crash. More uncertainty, including further cuts to Russia's oil exports, threatens greater turmoil in the second half of 2022.

While oil supply is shaky, oil demand remains bullish. The IEA predicts consumption will grow from 99 mb/d today to 103 mb/d later this decade. Though this is slow, compared to the yearly increases of the 20^th^ and early 21^st^ centuries – reflecting some degree of energy transition – demand is expected to maintain these record highs for decades. The IEA predicts, barring no radical shift in policies, these historic levels of consumption will be maintained until 2050.

Collectively, these experiences and estimations suggest it is far too premature to declare an imminent decline in oil demand. COVID has not altered oil's course as some predicted. It was a blip. There is, however, another side.

Oil demand may not be dying, but investment has been disturbed. Confronted by the uncertainty of the renewable energy transition, the volatile price climate, as well as problems in the increasing cost of new oil and gas production, the industry has had a difficult time mobilizing capital for future production. And, while COVID did not alter the trajectory of global oil demand, it did exacerbate this preexisting underinvestment into new production. In 2019, a little more than half a trillion dollars was invested in upstream oil production. In 2020, the pandemic cut $145 billion off this annual flow. In 2021 there was a slight increase, but investment remained $140 billion below what had been predicted without the pandemic (Rystad, 2021). This reduction from the pandemic, however, followed several years of record reductions in investment. In 2015 there was a 25 percent reduction in oil and gas capital flows, in 2016, a 24 percent reduction. Between then and 2020 there were only modest increases before, again, in 2020 there was a 29 percent reduction. These massive hits to investing in the future of oil production now total more than $400 billion annually, a reduction greater than the total amount invested in 2021. Essentially, global oil and gas investment has been cut in half over the last eight years. This is a historic downscaling. The decrease is so remarkable that the IEA sees (Figure 2) the cuts, and the current level of oil investment, as commensurate with their guidelines for achieving net-zero carbon emissions by 2050!

**Figure 2.** By author shows publicly available International Energy Agency (IEA, 2020) data on capital expenditures (USD, billions) for upstream oil and gas versus clean energy and infrastructure (renewables). The 2018-2030 Net Zero target is based on the IEA's Net Zero By 2050 report (IEA, 2021).

Superficially, this decline in oil investment is welcome. The catch, however, is that there is no commensurate rise in renewables. Hydrocarbon investments have been substantively down scaled – as if a net-zero transition was underway. But investments into renewable energy fall dramatically short of what would be required for that transition to materialize (see Figure 2). Some $1 trillion a year is invested in projects the IEA considers renewable – a figure which has not substantively changed in the last several years. However, they estimate investments must average almost $3.5 trillion per year for the next years, and then rise to $4 trillion per year in 2030, to achieve their net zero by 2050 carbon emissions scenario (IEA, 2021).

Recently, the economic geographer Brett Christophers (2021) has challenged the energy transition literature's focus on the falling price of renewables. While prices have fallen, profitability remains low. The capitalist energy transition, he suggests, must be understood through the lens of ‘future profitability’, i.e. capital accumulation. This framework is helpful for understanding this phenomenon of dual underinvestment into the future of energy. Prices for renewables have fallen, but the scale of investment required for a swift and decisive energy transition is not materializing because it would be unprofitable. Meanwhile, the fear and ongoing uncertainty in the oil industry – even now with higher prices – has prevented a commensurate boom in capital expenditures. Again, this emphasizes that price fluctuations are not the sole determinant of capital allocation. Amidst ongoing uncertainty over the future of the economy, the quality of new reserves, geopolitics and the renewable energy transition, a future boom is unlikely to entirely reverse the industry's losses. For example, most recently, the IEA (June, 2022) predicts that oil investments will rise slightly in 2022, however, they note this is ‘largely a reflection of higher costs’, not an acceleration of actual physical projects. This is not to claim that the oil industry is unprofitable – on the contrary, the oil companies reap record profits based on costs sunk long ago. Rather, it is to note that the dysfunctional investment dynamics, intrinsic to capitalist oil production, are, in this historic juncture of political, economic, and environmental crises, putting the future of energy into limbo.

The oil industry is, historically, both volatile and dysfunctional in its arrangement of private investment and market demand. Investment into oil is a long-term affair, heavy on upfront capital expenditure and light on ongoing costs – new production, largely, does not happen at the push of a button, but over years of exploring, planning, and drilling. Yet, when confronting glut, shutting down production hurts the long-term return from a field, as well as revenue streams whose costs have already been borne. Dating back to Standard Oil, the industry's history is interwoven with attempts to control the problems that result from these dynamics in the context of the commodity's private, anarchic, ownership (Yergin, 1991). Often these efforts are combined with spectacular violence aimed at disciplining unruly actors that disrupt an enforced tightness in the industry (Huber, 2011). The last 15 years, however, are one of the most volatile and dysfunctional periods of all the industry's bouts. The scale of this volatility exists on a day-to-day and month-to-month level as commodity trading houses speculate on the latest twists and turns of the market (see, this volume, Watts), including through profiting on intra-regional price variation and production bottlenecks. Volatility also persists in a larger cycle of multi-year booms and busts driven by stronger movements in investment, production and demand. The period 2003 to 2014 marked a gigantic boom, only briefly shocked by the 2008 downturn – between 2014 and 2020, a massive bust. Today, the tide would seem to have risen, but for how long, and at what cost? A new offshore field, costing in the billions of dollars, requires many years to just begin production. To ensure such a field's profitability, oil companies must predict prices ten to twenty years in the future. Amidst giant, ongoing booms and busts, the uncertain future of renewable energy policies, and the dangerous escalation of geopolitical tensions, oil investors are not eager to return to 2011 levels of capital expenditure. It is for this reason that – when adjusted for general inflation – investments are expected to remain at 2021 levels, despite roaring prices (IEA, 2022). Fearing years of volatile prices or the fleeting character of today's boom, producers are content to sit on their high rents, not risking being burned by the uncertain, anarchic future of their industry. The exception will be the highest quality (cheapest) projects that have not yet been invested in, for example, ExxonMobil's recent discovery in Guyana, and hydraulic fracturing in the United States. Fracking, unlike the rest of the industry, has a short-cycle materiality to it, giving it a contradictory advantage in the dysfunctional volatility of the industry (Eckhouse, 2021).

A type of rescaling, therefore, is proceeding in oil. However, it is a rescaling reflecting not the triumph of one energy over another, but the extremely contradictory character of capital accumulation at this unique juncture in energy production and global political economy.

Amidst confusion and concern about the future profitability of energy, global capital flows have been unable to decisively bet on *any energy future*. Capital flows to oil are so low, and the rates of decline in many developed fields so steep, the industry worries production will not keep at its current pace (Rystad, 2020). The allocated funds fall even further short of what would be needed to increase output to the expected demand increase in the coming years. Moreover, all these concerns existed prior to and independent of the conflict in Ukraine and the resultant questions over the future of Russia's exports. This rescaling is not a one-off restructuring of the oil and gas industry caused by COVID, but rather a more fundamental conundrum in the death of hydrocarbons. A conundrum which the pandemic has exacerbated. Market forces, themselves, are leading to – purely from the standpoint of price and investment – a dysfunctional energy transition, *even for big oil*. Severe, repeated gyrations in the price of hydrocarbons seem to be an inherent aspect of the capitalist energy transition. Oil's inherent tendency towards dysfunction and volatility is inflamed by today's conditions.

The importance of this conundrum – to a broad array of national, regional and local social processes – should not be overlooked. Already, in 2022, massive social protests have occurred over the rising cost of fuel and food, most notably in Sri Lanka. Sharp increases in the price of hydrocarbons cuts into the wages of billions of people. Blame can and should be placed on the massive oil companies – who themselves are reaping near record profits in 2022. Saudi Aramco's profits in the first quarter of 2022 surged 80 percent, for example, reaching $39.5 billion – Chevron's quadrupled, year over year. However, the more profound cause is not this or that company, but capitalism itself. Not only is the capitalist energy transition materializing too slowly to prevent severe climate change, but it is materializing in an anarchic way that threatens the livelihoods of billions. A feedback loop of unstable and chaotic energy prices is the result – hurting consumers, who, to no fault of their own, depend on oil.

This conception of the inherent market anarchy of the capitalist energy transition may be relevant to scholars working on sites and states bound to hydrocarbons. National and regional petro-economies (see Zalik, this volume) will have to contend with rescaling their operations in the face of instability. This will likely reward the cheap OPEC producers and the flexible fracking producers. In contrast, it will punish expensive tar sands, overly complex offshore, and floundering petrostates sorely lacking capital. Price increases and volatility may likewise influence the environment faced by activists and legal claimants in states with heavy, contested petroleum flows (see Lyall & Valdiva, this volume).

In conclusion, oil is not dying. It is stuck in a dangerous limbo of volatility and dysfunction. The contradiction of dual underinvestment into the future of energy points to the inability of capital accumulation, as a means of organizing labor, to enact a smooth energy transition. It is not that the substance ‘oil’ is preventing society from enacting a renewable energy transition. It is that the social and political economic relations through which society mobilizes labor to produce energy are, at present, inadequate, to rationally plan such a transition. The uncertainty and disruption caused by the pandemic has not pushed us closer to the end of oil. Rather, it has intensified this anarchic period of market confusion, the dysfunctional limbo. Now, the conflict in Ukraine, and the threat of a far more explosive, deadly world war, underscores the sustained role of oil and gas markets in the global economy. Demand for these fuels, however, will persist until the massive, trillions of dollars of investment, not currently forthcoming, are mobilized for a renewable future. The great need for a planned transition, run directly through democratic social control, not anarchic capital markets, directly raises the question of expropriating these companies and their financial backers on an international basis (see, in this volume, Huber).

## References

[bibr22-0308518X221114879] BridgeG, et al. (2013) Geographies of energy transition: Space, place and the low-carbon economy. Energy Policy. 10.1016/j.enpol.2012.10.066

[bibr23-0308518X221114879] Carbon Tracker (2020) Decline and Fall: The Size & Vulnerability of the Fossil Fuel System. https://carbontracker.org/reports/decline-and-fall/

[bibr24-0308518X221114879] ChristophersB (2021) Fossilised capital: Price and profit in the energy transition. New Political Economy. 10.1080/13563467.2021.1926957

[bibr25-0308518X221114879] EckhouseG (2021) United States Hydraulic fracturing’s short-cycle revolution and the global oil industry’s uncertain future. Geoforum; Journal of Physical, Human, and Regional Geosciences. 10.1016/j.geoforum.2021.07.010

[bibr26-0308518X221114879] Forbes (2020) “Despite Historic Production Cuts, Oil Is Dying,” Rapoza, K., April 15, 2020.

[bibr27-0308518X221114879] HuberM (2011) Enforcing scarcity: oil, violence, and the making of the market. Annals of the Association of American Geographers101(4): 816–826.

[bibr29-0308518X221114879] International Energy Agency (IEA) (2020) World Energy Outlook, 2020.

[bibr30a-0308518X221114879] International Energy Agency (IEA) (2021). Net Zero by 2050, May 2021.

[bibr30-0308518X221114879] International Energy Agency (IEA) (2022) World Energy Investment, June, 2022.

[bibr31-0308518X221114879] Jacobin (2020) The Oil Industry Is Dying Right Now. Don’t Resuscitate It.” Chris Saltmarsh, April 21, 2020, Jacobin. https://jacobinmag.com/2020/4/oil-barrel-price-crash-green-new-deal

[bibr32-0308518X221114879] Rystad (2020) Global investment slowdown set to hike oil prices and cause undersupply of 5 million bpd in 2025. May 4, 2020. https://www.rystadenergy.com/newsevents/news/press-releases/global-investment-slowdown-set-to-hike-oil-prices-and-cause-undersupply-of-5-million-bpd-in-2025/

[bibr6aa-0308518X221114879] Rystad (2021) Upstream spending, cut by $285 billion in two years, will struggle to recover to pre-pandemic levels. May 12, 2021. https://www.rystadenergy.com/newsevents/news/press-releases/upstream-spending-cut-by-$285-billion-in-two-years-will-struggle-to-recover-to-pre-pandemic-levels/

[bibr33-0308518X221114879] YerginD (1991) The Prize: The Epic Quest for Oil, Money and Power. New York: Free Press.

# Other: Trading houses & market volatility: Global commodity traders and the shifting landscape of oil and gas

**Authors:** Michael Watts2

**Affiliations:** 1438 University of California, Berkeley, USA; Swedish Collegium for Advanced Study, Uppsala, Sweden

The global commodity trading houses and the system within which they operate are crucial, yet understudied, in the broader transnational oil and gas assemblage (Blas and Farchy, 2021). Commodity trading is, in essence, a process of transforming commodities in space, time, and form. As intricate as it is opaque, the trading system is composed of different actors with contrasting forms of organizations, asset and ownership structures, interests and scale of operations within the commodity system. Physical trade – that is the purchase or sale of a particular kind or grade of product – and so-called paper trades (various forms of futures trades and option) are both essential parts of oil buying companies’ activities. Operating across multiple regulatory jurisdictions and a diverse transactional universe, commodity trading is one of the most baroque and difficult to regulate within the oil and gas global value chain (GVC) (Gillies, 2019). This system is moreover dynamic and adaptable. It has evolved, and is evolving, in relation to global capitalism in its recent financialized forms, and in response to market volatility and global competitive pressures. The restructuring of oil and gas markets nationally and internationally due to the global pandemic, and now, the war in Ukraine, underscore the need to deepen our understanding of this system (See Zalik and Eckhouse, this volume).

Several features contribute to making trading such a difficult segment of the GVC to regulate, notably its differentiation, dynamism and opacity. The trading system encompasses a suite of traders, intermediaries and enablers marked by a striking diversity of character, size, capability, and profile in their national economies (Gillies et al., 2015). International oil companies (IOCs) with their own trading desks sell their own product but also buy and sell third-party production. As ‘asset-based traders’ they have access to, and frequently use their own, capital to fund trading activities. BP, Shell and Total alone traded 15 million barrels of crude a day in 2016. In contrast, independent international commodity traders are generally companies that have not traditionally engaged in production, are often privately held, and typically deploy bank-provided trade finance to fund their activities. In 2019 the combined revenues of the ten largest traders was $1.4 trillion. Since 2010 there are also new entrants into the system from Russia, the UAE and China and a raft of smaller independent traders.

This system has emerged out of structural forces operating over four decades (Goldthau and Hughes, 2020; Moors, 2011; O'Sullivan, 2009). Global capitalism's thorough-going financialization triggered substantial changes in oil markets including the liberalization of oil trades in the 1980s and commodity indexes in the 1990s, all alongside the growth of futures contracts from new market actors (Gkanoutas-Leventis, 2017). The appearance of new actors was facilitated by permissive regulations such as the 2000 launch of the Commodities Future Modernisation Act (CFMA) in the US which opened oil commodity markets in general to mutual funds, hedge funds, insurance institutions, and, significantly, banks. The largest of these banks, known as ‘Wall Street Refiners’, established specialized departments for trading in the oil market (Gkanoutas-Leventis and Nesvetailova, 2015).

Traders and sellers are linked together in complex financial and joint-venture agreements that are diverse not only by virtue of the nature of the sale contracts and price negotiations, but also because of the elaborate networks linking companies, buyers, finance capital, audit houses and credit rating agencies (Guéniat et al., 2015). Over time, the large commodity traders have evolved into complex, multi-subsidiary, multi-jurisdictional organizations, often encompassing hundreds, if not thousands, of corporate entities linked by a complex web of ownership arrangements, marked by a high level of legal and geographical compartmentalization. Complex corporate arrangements make the identification of both the structure of trading houses and the function of its subsidiaries difficult to determine (Public Eye, 2017).

There is no common pattern among them in terms of the commodities they trade and transform, in the types of transformations they undertake, in their financing, nor in their forms of ownership (Ascher et al., 2012; Pirrong, 2014). Historically most traders do not have fully integrated supply chains and are ‘asset light’, chartering vessels and entering joint ventures with local counterparties. On a global canvas, much of the trading activity is clustered around a small number of global trading hubs and offshore financial centres [OFCs] in the UK, Netherlands, Singapore and Switzerland, in particular. Switzerland is the world's leading commodities trading hub with an estimated 35% share of the oil market. A 2017 report indicated that it housed over 500 trading companies. Almost 90% are private while 42% had less than 10 employees and 10% over 300 (IHRB, 2017). The largest independent firms are Glencore, Gunvor, Mercuria, Trafigura and Vitol. They vary in ownership structure and financial organization: Glencore is publicly owned and traded with significant shareholding by Qatar Investment, Blackrock and Harris Associates. Trafigura conversely is private owned by 700 senior employees, though with bonds held by GAM Holding, Close Brothers and Azimuth Investments, and underwritten by Standard Chartered, Bank of China and Sumitomo Mitsui.

In contrast to these firms, domestic buying companies operate only in the producing country and are heterogeneous in their scale and operations. Some are large and established, others are more akin to ‘middlemen’ or ‘briefcase’ companies often acting as intermediaries between the NOC and other larger buyers. National Oil Companies (NOCs), for the most part in the Global South, tend not to have their own sales, storage and distribution channels and therefore rely on oil and refinery companies, and independent traders. (Consider, in this regard, Lyall & Valdivia, this volume). To the degree they do have subsidiaries that trade in commodities they typically trade in their own oil. Finally, there are investment banks that trade commodities such as Goldman Sachs and Citigroup – the number of which has fallen dramatically due to bank regulatory changes after 2008 and by the market risks surrounding the 2014 price collapse – and the end-users such as refiners, smelters and processors (e.g. Sinopec, Japanese refiners such as JX Nippon Oil and Energy Corp.).

All traders depend upon liquidity and loans, typically through a suite of instruments to manage financing and settling of accounts. This often includes complex and opaque arrangements. First trades are often associated with a particular class of risks associated with resource-backed loans (RBLs), namely loans provided to a government or a state-owned company where repayment is either made directly in natural resources, or repayment is guaranteed by a natural resource-related income stream, or a natural resource asset serves as collateral. RBLs are simply one set of transactions linking buyers and NOCs but they carry significant risks because of their magnitude, duration and opacity (Sayne and Gillies, 2016). NRGI's analysis reveals how RBLs carry immense major public finance risks reflected in the fact that of 14 RBL recipient countries, ten experienced serious debt problems after the commodity price crash of 2014 with RBLs often an important contributor (Mihalyi et al., 2010).

A core attribute of the trading system is the role played by commodity trading hubs and offshore financial centres (OFCs). The main strategic trading hubs historically have been located in the US (Houston, Chicago) and Europe (London, Amsterdam and Geneva/Zug) but the landscape is dynamic and recently there has been a clear ‘drift eastwards’ in the direction of Dubai, Singapore, Shanghai, and Hong Kong. Independent traders make extensive use of offshore financial centre jurisdictions. A sample of the largest independent trading houses reveal that 96.7% make extensive use of OFCs (Nesvetailova et al., 2021). While OFCs can have well developed regulatory institutions, they are increasingly under scrutiny for facilitating tax avoidance and having laws and systems that are hostile to transparency. Opacity and secrecy are compounded by the fact that traders may use intermediaries, further complicating the picture. In Nigeria, for example, several beneficiaries of export allocations are nothing but letterbox companies whose sole merit is that they are linked to high-ranking political officials or their entourage.

To conclude, I turn to the impact of COVID (and related financial crises), and the transitions to low carbon economies. Two recent trends are of note. The first is with respect to market volatility and physical asset intensity. Commodity trading turns on high volume low-margin arbitrage. But in the period after the financial crash of 2008/9, falling tradition margins encouraged private trading firms to expand into other parts of the supply chain (storage and distribution, refining) (Baines and Hager, 2021). The top four oil traders accounted for 84 acquisitions between 2010 and 2019 (compared to 12 between 1990 and 1999). At the same time, they generated less income from their financial divisions, some of which were closed. The second notable trend, stemming from bank regulatory changes in the wake of the 2008/9 financial crisis, was that major investments houses and banks withdrew from their traditional role in providing liquidity to the trading firms. The demand for liquidity did not change but the financial architecture has. As Public Eye (2020) has documented, the firms now resort to risky open account payments, revolving credit facilities involving banking syndicates, non-bank financing through bonds and largely unregulated private equity, and by traders assuming the role of bankers through oil-backed loans and oil-swaps. The volume of such corporate and revolving credit facilities is substantial. In some cases, finance appears to be derived from regional banking syndicates about which little is known. Such changes in asset-structure raise concerns about the risk of illicit financial flows and the opacity of trader practice.

Against the backdrop of global crises and financial volatility, the trading system has been shown to be adaptable, dynamic and changing (Seddon, 2020). The wariness of major investment banks to fund trading has in fact facilitated the growing dominance by the big trading houses and the IOC trading desks. Meanwhile, smaller traders have suffered. Market volatility, whether a product of COVID, financial crises or other contingent events (currently the Russia-Ukraine crisis), is the bread and butter of commodity trade success. While traders cannot ignore the pressures of renewable energy on the oil sector, these same pressures may, in fact, be the source of future volatility and thus profits (see Eckhouse, this volume). In the meantime, the further expansion of LNG and the intensification of market volatility following the further breakdown of the post-war order, contribute to the bullish atmosphere in the world's commodity trading hubs.

## References

[bibr66a-0308518X221114879] Ascher J, Lazo P and Quiviger G (2012) *Commodity Trading at a Strategic Crosssroads*. McKinsey & Company. https://www.mckinsey.com/∼/media/mckinsey/dotcom/client_service/risk/working%20papers/39_%20commodity_trading_at_a_strategic_crossroad.ashx

[bibr34-0308518X221114879] BainesJHagerSB (2021) Commodity traders in a storm: Financialization, corporate power and ecological crisis. Review of International Political Economy28: 1–55.

[bibr35-0308518X221114879] BlasJFarchyJ (2021) The World for Sale: Money, Power, and the Traders who Barter the Earth’s Resources. London: Oxford University Press. 2021.

[bibr36-0308518X221114879] GilliesA (2019) Crude Intentions: How oil Corruption Contaminates the World. New York: Oxford University Press.

[bibr37-0308518X221114879] GilliesASayneAKatsourisC (2015) Inside NNPC Oil Sales: A Case for Reform in Nigeria. New York: Natural Resource Governance Institute.

[bibr38-0308518X221114879] Gkanoutas-LeventisA (2017) Spikes and Shocks: The Financialization of the oil Market from 1980 to the Present day. London: Palgrave.

[bibr39-0308518X221114879] Gkanoutas-LeventisANesvetailovaA (2015) Financialisation, oil and the great recession. Energy Policy86: 891–902.

[bibr40-0308518X221114879] GoldthauAHughesL (2020) Saudi on the rhine? Explaining the emergence of private governance in the global oil market. Review of International Political Economy128/5: 1410–1432.

[bibr41-0308518X221114879] Guéniat et al. (2015) Philia’s Refined Ventures in Brazzaville – How Swiss Traders Misappropriate Congolese Oil Rents, Berne Declaration. https://www.publiceye.ch/fileadmin/doc/Rohstoffe/2015_PublicEye_Philia_s_Refined_Venture s_EN_Report.pdf.

[bibr76a-0308518X221114879] IHRB (2017) *The Swiss Commodity Trading Industry*. Zurich: Institute for Human Rights and Business. https://www.ihrb.org/uploads/reports/IHRB%2C_The_Swiss_Commodities_Trading_Sector_-_A_Mapping_Study%2C_Mar_2017.pdf

[bibr42-0308518X221114879] MihalyiDAdamAHwangJ (2010) Resource-Backed Loans: Pitfalls and Potentials. New York: NRGI.

[bibr43-0308518X221114879] MoorsK (2011) The Vega Factor: Oil Volatility and the Next Global Crisis. New York: Wiley.

[bibr44-0308518X221114879] NesvetailovaAPalanRPetersenH, et al. (2021) “Workstream 2 Report: IFFs and Commodity Tradings -- Mapping Networks of Corporate Arbitrage in Oil and Gas Trading, University of London, London.

[bibr45-0308518X221114879] O’SullivanD (2009) Petromania: Black Gold, Paper Barrels and oil Price Bubbles. London: Harriman House Limited.

[bibr46-0308518X221114879] PirrongC (2014) The Economics of Commodity Trading Firms. Trafigura: Zurich. (https://www.trafigura.com/media/1192/2014_trafigura_economics_of_commodity_trading_firms_en.pdf)

[bibr47-0308518X221114879] Public Eye (2017) Oil and Embezzlement: Public Eye Reveals Gunvor’s Secrets in Congo, Public Eye. https://www.publiceye.ch/en/media-corner/press-releases/detail/oil-and-embezzlement-public-eye-reveals-gunvors-secrets-in-congo/;

[bibr48-0308518X221114879] Public Eye (2020) Trade Finance Demystified. Lausanne: Public Eye. Public Eye (2018) Vitol, the King of Oil in Kazahkstan, Public Eye.

[bibr49-0308518X221114879] SayneAGilliesA (2016) Initial Evidence of Corruption Risks in Government Oil and Gas Sales."Natural Resources Governance Institute, New York Retrieved fromhttps://resourcegovernance. org/sites/default/files/documents/nrgi_trading-corruption-risk. pdf.

[bibr50-0308518X221114879] SeddonJ (2020) Merchants against the bankers: The financialization of the commodity market. Review of International Political Economy27/3: 525–555.

# Other: On oil capitals and empire: The advance of international arbitration under COVID-19

**Authors:** Angus Lyall, Gaby Valdivia

**Affiliations:** 27902 Universidad San Francisco de Quito, Quito, Ecuador; University of North Carolina, Chapel Hill, North Carolina, USA

## Introduction

Ecuador was the site of one of the most well-known lawsuits in the world to address human- rights violations by a transnational oil company (Pellegrini et al., 2020; Valdivia, 2007). In 1993, 30,000 plaintiffs filed suit against oil-giant Texaco for failure to remediate contamination during its 30-year concession in the Ecuadorian Amazon. In 2001, Chevron acquired Texaco – and its legal obligations – and nearly a decade later, after numerous site inspections, a series of appeals and counter appeals, and intense media attention, the plaintiffs won. Pablo Fajardo, the Ecuadorian lawyer in the case, proclaimed that this historic moment signaled that multinational corporations were no longer ‘untouchable’. However, Chevron refused to pay the $9.5 billion award. In a practice that legal experts call ‘forum shopping’, the company sought a favorable ruling in international arbitration. Citing the bilateral investment treaty (BIT) between Ecuador and the United States, which granted jurisdiction for commercial disputes to The Hague's Permanent Court of Arbitration, the US-based Chevron moved the trial to The Hague. There, outside of public scrutiny, arbiters ruled in an astonishing twist that the Ecuadorian state compensate Chevron.

The outcome of this trial should be understood in the context of developments in international treaties over the last half century. Since the first bilateral investment treaty (BIT) between West Germany and Pakistan in 1959, BITs have included international arbitration clauses, which ensure that transnational companies can have their disagreements with states adjudicated in confidential venues, beyond the reach of national jurisdiction. BITs emerged in the historical context of the decolonization of much of the global South, as imperial forces withdrew from colonies but transnational companies sought to retain control over natural resources, particularly oil (Lustig, 2020). In effect, BITs have enabled transnational companies to take their host countries to court instead of submitting themselves to host country law, extending privileges to foreign companies and restricting state sovereignty. In this way, BITs have become a vehicle for creating a new form of international law called ‘transnational law’ or ‘private international law’, a key innovation for accelerating the globalization of capital. By 2000, Michael Hardt and Antonio Negri (2000) observed that global capital flows relied on a network of institutions (e.g. WTO, IMF, World Bank) and practices – which these authors coined “Empire” – that secure capital investments, in part through the codification and promotion of transnational law. In 1956, The International Chamber of Commerce's International Court of Arbitration received just 32 cases, but in 1976 it received 210 new cases (Ruiz, 2011). The World Bank's International Centre for Settlement of Investment Disputes (ICSID) received two cases annually from 1972 to 1996, but in the 2000s this increased to 24 cases annually and in the 2010s the average jumped to 50 cases annually (ICSID, 2021). Recently, Hardt and Negri (2019) noted that the rise of nativists and nationalists in global politics contrasts with the inconspicuous entrenchment of global institutions of capital regulation, bolstered by the proliferation of nearly 3000 BITs worldwide.

91% of claimants at The World Bank's International Centre for Settlement of Investment (ICSID) are from the global North (Ruíz, 2020). In the first decade of the 2000s, various governments in the global South resisted this mechanism of neoliberal globalization. South Africa, Indonesia, Bolivia and Venezuela, among other states, withdrew from BITs. The nationalist regime of Ecuadorian president Rafael Correa (2007–2017) eliminated BITs and struck arbitration clauses from new contracts. Such resistance, however, proved ephemeral. Today, neoliberal regimes are renewing commitments to arbitration to attract oil capitals and secure them against social, political, and economic volatility. In this brief intervention, we examine how ruling elites in Ecuador are leveraging disillusionment with national institutions in order to re-commit national oil reserves to international arbitration and restrict sovereign control over oil flows into the future.

## International arbitration and Ecuadorian oil

Transnational oil companies and their networks of political, commercial and financial intermediaries – often characterized by their opacity (Watts, *this issue*) – have consistently insisted on international arbitration clauses in their contracts in order to conceal future disputes from the public and protect themselves from contractual or regulatory changes. Relatively minor disputes over accounting or the calculation of recovery costs are common over the lifespan of oil contracts, but arbitration is also used in more contentious disputes involving social conflict or price volatility. For example, environmental or human rights concerns might place pressure on a state to terminate a contract or change regulations and price volatility might cause states to fail to make payments. But arbitration clauses discourage states from breaking contracts or re-regulating industries insofar as foreign firms can demand compensation to neutralize impacts.

Historically, transnational companies in Latin America and the Caribbean have won just 50% of the cases they have brought before the ICSID and, in Ecuador, the state has only been found *not liable* in 22.5% of proceedings (Ruíz, 2020). Thus, the 2008 Constitution of Ecuador sought to challenge this mechanism of capital regulation by prohibiting the state from ratifying treaties ‘in which the Ecuadorian state cedes sovereign jurisdiction to entities of international arbitration, in contractual controversies or of commercial nature’ (195, *translation ours*). This constitution only permits arbitration *within* Latin America. In the wake of the constitutional assembly, President Rafael Correa withdrew from the ICSID and denounced BITs as unconstitutional – an argument validated by the Constitutional Court in 2010. In this context, multiple oil investors were dissuaded from investing in Ecuador by ongoing social resistance in the Amazon and a poor track-record of state payment to firms (Valdivia and Lyall, 2018).

The Correa government did acquiesce to a series of unfavorable arbitration decisions from prior contracts. By late 2020, Ecuador had accumulated 69 cases against it (Jijón et al. 2021: 97) and to-date the Ecuadorian state has complied with *all* rulings – even in costly, public disputes with Occidental Petroleum and Chevron – to sustain its image as a ‘compliant state’.

Yet, since 2021, President Guillermo Lasso has sought the attention of investors by openly and actively embracing arbitration for future contracts with transnational companies. Specifically, Lasso requested that a new cohort of judges on the Constitutional Court enable Ecuador to return to the ICSID. His administration was given a green light and Lasso promptly signed the corresponding decree. His government is now awaiting a decision by the same court that might formally permit new BITs. The oil and gas sector has read these gestures optimistically. For example, the CEO of the small firm Wayra Energy, which has increased investment in Ecuador in recent years from $50 to $300 million USD, explains his enthusiasm: ‘Back in 2017, investment funds were quite reluctant to grant financing for projects in Ecuador. However, the new administration started to take steps to become more attractive for investment… Investors first seek to have efficient ways of resolving possible conflicts using international arbitration tribunals being appointed under contractual terms’ (*TheBusiness Year*, 2020).

We observe that this shift back towards international arbitration in Ecuador corresponds with rising political disillusionment in Ecuador and across the globe. ‘Political disillusionment’ refers to increased skepticism regarding the capacities and stated-intensions of elected leaders and public institutions. Disillusionment has risen in diverse contexts in recent years – exacerbated by the COVID-19 pandemic, in effect increasing voter apathy and political polarization (Barberia et al., 2021). This sort of disillusionment is certainly not new in Ecuador. The Right remains stained by a tumultuous decade of neoliberal reforms and protests (1992–2006) that led to the rise and fall of seven presidents. The institutional Left – in power from 2007 to 2017 – lost credibility with the collapse of commodity prices in 2014 and the revelation of corruption (Lyall, 2018). Yet, disillusionment in electoral politics and public institutions has risen notably since 2020. In the national elections of 2021, the ‘null’ or ‘no candidate’ vote nearly tripled as compared to the elections of 2017, reaching its highest level since Ecuador's return to democracy in 1978. That is, the narrow victory of Lasso over Correa's chosen successor cannot be attributed to a shift to the Right, but rather a rise in disillusionment (Rodríguez, 2021). In this context, Lasso has continuously attacked public programs and institutions, advancing an IMF-endorsed, neoliberal agenda of austerity, privatization, and trade liberalization (Vela- Almeida et al., 2021). By the same measure, he has advanced the legitimacy of international actors in Ecuadorian affairs, from security to trade policy, harping the slogan ‘We need more Ecuador in the world and more world in Ecuador’. The Indigenous movement has attempted to resist neoliberal reforms through nationwide uprisings in 2019 and 2022, but international arbitration has not been one of its targets. In this milieu, international arbiters are poised to re- establish their authority in Ecuadorian oil governance.

Since the 1970s, global labor standards have eroded in what has been widely described as a ‘race to the bottom’, as states in the global South have stripped away regulations to attract foreign investment. The same phrase might adequately describe the resolution of disputes between states and transnational companies. Dispute resolution has increasingly favored capital through the erosion of sovereign governance and expansion of international arbitration. Looking ahead, energy transitions across the globe are bound to entail oil price volatility (Eckhouse, *this issue*); social pressure to re-regulate oil production or expropriate oil to leave it underground (Huber, *this issue*); and geopolitical conflicts (Zalik, *this issue*) – each of which will spark disputes between states and transnational companies. Today, amid deepening disillusionment with national institutions, oil investors, firms, and politicians quietly continue to erode national capacities to govern oil into what is sure to be a contentious future.

## References

[bibr51-0308518X221114879] BarberiaLPlümperTWhittenG (2021) The political science of COVID-19: an introduction. Social Science Quarterly102(5): 2045–2054.

[bibr52-0308518X221114879] Constitution of the Republic of Ecuador (2008) (translation ours). Available at: www.turismo.gob.ec/wp-content/uploads/2021/09/Constitucion-de-la-Republica.pdf. (accessed 10 March 2022).

[bibr53-0308518X221114879] EckhouseG (This edition) COVID, global oil market volatility, and the renewable energy transition. Environment and Planning A 1–27.

[bibr54-0308518X221114879] HardtMNegriA (2019) Empire, twenty years on. New Left Review (120): 67–92. https://newleftreview.org/issues/ii120/articles/empire-twenty-years-on.

[bibr55-0308518X221114879] HardtMNegriA (2000) Empire. Cambridge, MA: Harvard University Press.

[bibr56-0308518X221114879] HuberM (This edition) Expropriating hydrocarbons in the 21st century: Lessons from history. Environment and Planning A 1–27.

[bibr57-0308518X221114879] International Centre for Settlement of Investment Disputes (ICSID) (2021) The ICSID Caseload — Statistics. Available at: www.icsid.worldbank.org/sites/default/files/Caseload20Statistics20Charts/The20ICSID20Caseload20Statistics202021-220Edition20ENG.pdf (accessed 10 March 2022).

[bibr58-0308518X221114879] Jijón LetortRMarchánJJaramilloJ (2021) The Ecuadorian approach to international arbitration. In: AllenJSouH (eds) The Arbitration Review of the Americas 2022. New York: Global Arbitration Review, pp.95–101. Available at:www.pbplaw.com/sitio/wp-content/uploads/2021/INSIGHTS/PBP-global-arbitration-review-2022.pdf (accessed 10 March 2022).

[bibr59-0308518X221114879] LustigD (2020) Veiled Power: International Law and the Private Corporation 1886–1981. Oxford, UK: Oxford University Press.

[bibr1000-0308518X221114879] LyallA (2018) A moral economy of oil: Corruption narratives and oil elites in Ecuador. *Culture*. Theory and Critique59(4): 380–399.

[bibr60-0308518X221114879] PellegriniLArselMOrta-MartínezM, et al. (2020) International investment agreements, human rights, and environmental justice: The Texaco/Chevron case from the Ecuadorian Amazon. Journal of International Economic Law23(2): 455–468.

[bibr61-0308518X221114879] RodríguezJ (2021) El voto nulo y el triunfo de la derecha en Ecuador. Nueva Sociedad293: 4–13.

[bibr62-0308518X221114879] RuízG (2020) Who wins in international investment arbitration disputes? Evidence from Latin American and Caribbean countries cases under ICSID. Available at SSRN: www.ssrn.com/abstract=3842581 or 10.2139/ssrn.3842581 (accessed 10 March 2022).

[bibr63-0308518X221114879] RuizF (2011) Regulating trade, investment and money. In: CrawfodJKoskenniemmi (eds) The Cambridge Companion to International Law. Cambridge, UK: Cambridge University Press, pp. 352–372.

[bibr64-0308518X221114879] The Business Year (2020) Successful bidder in oil rounds. Available at: https://www.thebusinessyear.com/ecuador-2020/successful-bidder-in-oil-rounds/interview (accessed 10 March 2022).

[bibr65-0308518X221114879] ValdiviaG (2007) The “Amazonian trial of the century”: Indigenous identities, transnational networks, and petroleum in Ecuador. Alternatives32(1): 41–72.

[bibr66-0308518X221114879] ValdiviaGLyallA (2018) The oil complex in Latin America: Politics, frontiers, and habits of oil rule. In: CupplesJPalomino-SchalschaMPrietoM (eds) The Routledge Handbook of Latin American Development. New York: Routledge, pp. 458–468.

[bibr67-0308518X221114879] Vela-AlmeidaDLyallALassoG, et al. (2021) Resisting austerity in the era of COVID-19: Between nationwide mobilisation and decentralised organising in Ecuador. In: CalvárioRKaikaMVelegrakisG (eds) The Political Ecology of Austerity: Crisis, Social Movements, and the Environment. New York: Routledge, pp. 135–155.

[bibr68-0308518X221114879] WattsM (This edition) Trading houses and market volatility: Global commodity traders and the shifting landscape of oil and gas. Environment and Planning A 1–27.

[bibr69-0308518X221114879] ZalikA (This edition) The perpetual rescaling of oil and gas production and flows in continental North America.

# Other: The perpetual rescaling of oil and gas production and flows in continental North America

**Authors:** Anna Zalik

**Affiliations:** 7991 York University, Toronto, Canada

In its January 2022 Global Economic Outlook *Export Development Canada (EDC)*, Canada's federal investment bank, points to the inflation and price increases arising from pandemic induced supply chain constraints. They strike an optimistic note:

Higher prices are the best remedy for higher prices. Greater profit margins spur higher supply, either from existing businesses or new entrants eager to cash in on the good times. Thankfully, this dynamic is already leading to an increase in supplies of needed goods and services, spurring some to speculate that the worst is over.^3^

This is a marked contrast to 2020. In March of that year, just weeks into the first Covid lockdown, the Canadian Association of Petroleum Producers sent a memo to Canada's Minister of Natural Resources requesting regulatory rollbacks while thanking the federal government for initiating a ‘Covid 19 Market Crisis Joint Working Group’.^4^ In April 2020, EDC announced extra supports to Canadian oil and gas producers including a Reserve Based Lending guarantee to keep operators afloat.^5^ This occurred during mergers and acquisitions of firms significantly held by US and international capital (Laxer, 2021) among them Cenovus by Husky, Seven Generations by ARC resources and Painted Pony by Canadian Natural Resources Ltd. Notably Shell proceeded with its strategy of ‘divesting’, ‘non-core’ assets, in this case its Duvernay shale fields to Crescent Point Energy.

EDCs injection of capital to support Canada's oil and gas sector under pandemic conditions was far from unprecedented. EDC has offered significant financing over the past decade to what might be understood as a scalar shift in continental energy supply: Canadian tar sands producers, and the pipeline companies that move their product, have faced increasing activist pressures and price volatility (see Eckhouse this section) – making costly and CO_2_ intensive bitumen a less attractive financial investment. Concurrently, EDC has underwritten growing Canadian interests in the denationalization and restructuring of the Mexican energy sector. EDC has also supported infrastructural projects in Canada aimed at shipping bitumen sands to sites in Asia where climate-activist pressures against tar sands have had less impact than in Europe. Although such projects, notably in Liquefied Natural Gas (LNG), stalled under COVID due to pressures on the global shipping industry, supply constraints in Europe arising from the war in Ukraine have led to a resurgence in demand for global LNG shipments.

In Canada, one outcome of this most recent interest in LNG are moves to complete contentious LNG infrastructure on both the West and East coast. Notable here is the British Columbia *Coastal Gas Link (CGL)* pipeline supplying the *LNG Canada* terminal, a project opposed by Wet'suwet’en hereditary chiefs whose resistance to the project was supported through the cross- country ‘Shut down Canada’ (McCreary, 2020; Pasternak and Dafnos, 2018; Wilson, 2020) actions of February 2020. Occurring just prior to the first Covid lockdown, this movement effectively blocked Canada's rail system^6^. Pertinent to the analysis here, TC Energy (formerly TransCanada pipelines, the progenitor of the Keystone XL pipeline)^7^ is the CGL pipeline operator, a firm that holds contracts for a major pipeline network fueling Mexico with US shale gas – and whose activities have received significant backing from the Canadian government, notably via EDC financing. Also salient, LNG Canada is operated by Shell – a firm that divested from Alberta's tar sands operations following a successful Indigenous-led mobilization against an attempted expansion of its Jackpine mine.

The use of Coastal Gas Link as project and firm name, has allowed prominent operators Shell and TC Energy to shield themselves from negative social pressures and financial risk assessment. Such naming strategies hedge reputational and financial risk, part of the protection tactics promoted via what Seabrooke and Wigan (2017) describe as ‘Global Wealth Chains’. Firms such as TC Energy offset protest and regulatory blocks for projects such as the Keystone XL pipeline via movement to and investment in Mexico, and in parallel seek to shed the negative reputation of Canadian extraction in Latin America through a name change. Until 2020 known as TransCanada pipelines, TC Energy – which renamed itself shortly after the first Covid lockdown when attention to it was limited by pandemic concerns - is now described as the largest Canadian investor in Mexico.^8^

Pandemic dynamics and associated supply chain anxieties have thus armored the ongoing shifts in continental energy systems and continental politics to secure oil and gas projects and markets for their products. Such shifts in North America are built atop a significant rescaling in hydrocarbon flows in the five years prior to covid, one striking in its reversal of internal relations within the NAFTA bloc (Saxe Fernandez, 2016; Vargas, 2014). Central to this rescaling was the transformation of the US into a net exporter rather than net importer of hydrocarbons from/to its NAFTA (now CUSMA/TMEC/USMCA) partners. The US shale boom was a key factor in this shift, a boom itself the consequence of conditions equally continental/regional as national and global: 1) the push in the US to reduce imports of supplies from Saudi Arabia and other gulf producers following 9/11, 2) the pressure to end investment in the Alberta tar sands due to their exorbitant C02 emissions, ecological impact and a global campaign against Canada's violations of Indigenous territorial rights and 3) the intensified denationalization of Mexico's energy sector via a 2014 reform bill that drastically accelerated the privatization of that country's petroleum industry. It is out of these co-constituted conditions that Canada's and Mexico's role at the turn of millennium as fuelers of ‘fortress America’ (Gutiérrez-Haces, 2015; McCullum, 2006) were similarly inverted. Mexico became increasingly dependent on US fracked gas, supplied through pipelines such as those constructed by TC Energy, to feed its energy grid (Hernandez Cervantes and Zalik, 2018). In response to these shifts and in the face of restrictions on energy autonomy that persist in the NAFTA 2.0 agreement, the Andres Manuel Lopez Obrador (AMLO) Morena government (elected in part in reaction to the country's loss of energy sovereignty) proposed a partial reversal via a 2022 energy reform bill rejected by the government's opponents in the Mexican congress. Criticized by the US and Canada, if it had passed the legislation would have fomented a return to greater national energy autonomy in Mexico (see also Huber this section), which AMLO has nevertheless pursued through a bill protecting lithium as a nationalized resource (Nunez Rodriguez, 2022).^9^

The importance of terrestrial or spatially proximate hydrocarbon access to energy security ties domestic energy policy, including with relation to controversial infrastructure such as pipelines and critical minerals, to continental geopolitics (Bridge et al., 2018; Temper et al., 2020). In North America, global volatility in oil and gas markets is ensconced in the regional market regulations of NAFTA – now CUSMA/TMEC/USMACA (MacDonald, 2019).^10^ Under the NAFTA 2.0 agreement signed as the new Mexican government entered office, Canadian oil production was freed from the NAFTA ‘proportionality clause’ that committed it to maintain, and not reduce, the share of production it directed at US markets. Intended, in the initial NAFTA agreement, to protect US energy security that clause became redundant due to the transformation in continental energy production and resource geopolitics described above (Dillon, 1993). While never invoked, the proportionality clause threatened to restrict Canada's ability to reduce hydrocarbon exports to the US, potentially blocking Canadian domestic strategies to use oil and gas production domestically as a first step towards transitioning off fossil fuels. And whereas the US and Canada have also been relieved of a controversial ‘investor state’ clause (see also Lyall & Valdivia this section) under NAFTA 2.0 that had allowed private companies to sue should subsequent environmental or social regulations impede their contracts, Mexico remains beholden to these pressures in the energy sector, discouraging a potential renationalization of its oil and gas industry. This threat of litigation against Mexico expresses how capital's use of legal mechanisms (ibid) blocks anti-imperialist tendencies within Mexico's resource politics, tendencies deeply tied to a discourse of national energy sovereignty embedded in Mexico's 1917 revolutionary constitution.

From this vantage point, the crux of this intervention is at once empirical and methodological. The most recent significant rescaling of the North American hydrocarbon market predates Covid. While often attributed to the US shale boom, it was co-constituted alongside underinvestment in the Mexican energy sector and a decline in Mexican energy sovereignty, alongside rising protest against tar sands in development Canada (Adkin, 2015; Carter, 2020). From the dominant vantage point of methodological nationalism, the outcome of this process is described as facilitating the rise of the US as an energy superpower. It is associated, culturally with (often reactionary) pro-oil ideologies across the continent, alongside a contrasting progressive critique of global capital centring the importance of domestic energy sovereignty and autonomy (Laxer, 2021; Observatorio de la Energia, 2021) within spatially linked market blocs. Indeed, a national governance project remains the most practicable means for bringing about the massive infrastructural change entailed by an energy transition from hydrocarbons (Huber this section)^11^. This view is central to the recent political developments in Mexico, notably the April 2022 bill to guarantee national control over lithium.

In contrast to methodological nationalism, however, supply constraints under Covid highlight the need for a unit of analysis centred on spatially connected, often continental, regions in hydrocarbon trade circuits, including their constitution through global wealth chains (see also Lesage and Van de Graaf, 2016). Recent geographical scholarship concerning the crucial role of midstream actors in global production networks (Bridge and Bradshaw, 2017; Watts this section) is further enriched through attention to the regional political economic structures of trade agreements, transcontinental firm strategy, and the global legal architectures (Lyall & Valdivia, this section) that accompany them. Indeed, combining a continental unit of analysis with attention to national governance and domestic energy politics helps scholars consciously de-reify the state and its spatial boundaries, while still attending to the real effects of state institutions. And, as emphasized herein, a continental scope allows us to see how the rescaling of hydrocarbon flows within transnational, terrestrially connected regions is co-constituted with the re-regulation of world trade in the covid conjuncture – one that comprises the most significant transformation in global economic regulation seen in at least the past half century.

## References

[bibr70a-0308518X221114879] AdkinL (ed.) (2016) First World Petro-Politics: The Political Ecology and Governance of Alberta. University of Toronto Press.

[bibr70-0308518X221114879] BridgeGBradshawM (2017) Making a global gas market: Territoriality and production networks in liquefied natural gas. Economic Geography93(3): 215–240.

[bibr70b-0308518X221114879] BridgeGÖzkaynakBTurhanE (2018) Energy infrastructure and the fate of the nation: Introduction to special issue. Energy Research & Social Science41: 1–11.

[bibr86a-0308518X221114879] Carter A (2020) *Fossilized: Environmental Policy in Canada's Oil Provinces*. Vancouver: UBC Press.

[bibr72-0308518X221114879] CervantesAHZalikA (2018) Canadian Capital and the denationalization of the Mexican energy sector. Journal of Latin American Geography17(3): 42–72.

[bibr73a-0308518X221114879] DillonJ (1993) The petroleum sector under continental integration. In: GrinspunRCameronMA (eds) The Political Economy of North American Free Trade. London: Palgrave Macmillan, pp.3–25.

[bibr971-0308518X221114879] EckhouseG (this section) COVID-19, global oil market volatility, and the renewable energy transition.

[bibr71-0308518X221114879] GosdenE (2022) Commodities firms continue trading in Russian oil and gas. Times of London: March 12.

[bibr73b-0308518X221114879] Gutiérrez-HacesMT (2015) Los Vecinos del Vecino. La continentalización de México y Canadá en América del Norte. Ciudad de México: Universidad Nacional Autónoma de México/ARIEL.

[bibr73c-0308518X221114879] HuberM(this issue) Expropriating hydrocarbons in the 21st century. Economy and Space.

[bibr73-0308518X221114879] LaxerG (2021) Posing as Canadian: How Big. Foreign Oil Captures Canadian Energy and Climate Policy. Council of Canadians/Canadian Centre for Policy Alternatives.

[bibr74ab-0308518X221114879] LesageDVan de GraafT (2016) Global Energy Governance in a Multipolar World. Routledge.

[bibr74abc-0308518X221114879] Lyall and Valdivia (this issue) On oil capitals and empire: The advance of international arbitration under COVID-19. *Economy and Space*.

[bibr73ab-0308518X221114879] MacdonaldL (2019) Upsetting the apple cart? Implications of the NAFTA re-negotiations for Canada–US relations. In:Canada–US Relations. Palgrave Macmillan, pp.193–213.

[bibr74-0308518X221114879] McCullumH (2006) Fuelling Fortress America: A Report on the Athabasca Tar Sands and US Demands for Canada’s Energy. CCPA, Parkland, Polaris, Ottawa.

[bibr75ab-0308518X221114879] McCrearyT (2020) Between the commodity and the gift: the Coastal GasLink pipeline and the contested temporalities of Canadian and Witsuwit’en law. In:From Student Strikes to the Extinction Rebellion. Edward Elgar Publishing, pp.122–145.

[bibr73bb-0308518X221114879] NunezRodriguez V (2022) La batalla por el litio de México. Mexico, Editorial Entretejas.

[bibr75-0308518X221114879] Observatorio de la Energia (2021) Y Por Que No Mejorar PEMEX? Editorial Marzo 2021.

[bibr75aaab-0308518X221114879] PasternakSDafnosT (2018) How does a settler state secure the circuitry of capital?Environment and Planning D: Society and Space36(4): 739–757.

[bibr76-0308518X221114879] SeabrookeLWiganD (2017) The governance of global wealth chains. Review of International Political Economy24(1): 1–29.

[bibr76bbc-0308518X221114879] Saxe FernandezJ (2016) La Compraventa de Mexico: Una interpretación histórica y estratégica de las relaciones México-Estados Unidos. UNAM/CEIICH, Mexico.

[bibr76bb-0308518X221114879] Temper L,AvilaS, DelBene D, et al. (2020) Movements shaping climate futures: A systematic mapping of protests against fossil fuel and low-carbon energy projects. Environmental Research Letters15(12): 123004.

[bibr77ab-0308518X221114879] VargasR (2014) El papel de México en la integración y la seguridad energética de Norteamérica. Mexico: CISAN/UNAM.

[bibr77-0308518X221114879] WilsonL (2020) Shut down Canada’: Gidmit’en spokesperson calls for people to take to the streets. APTN NEWS. February 7.

# Other: Expropriating hydrocarbons in the 21^st^ century

**Authors:** Matt Huber

**Affiliations:** 2029 Syracuse University, Syracuse, USA

## Introduction

Nearly two years after the COVID collapse, boom times have returned to the oil industry. Amid skyrocketing profits, production is ramping up in the Permian Basin (McCormick, 2022) and OPEP + countries have also agreed to raise output (Meredith, 2022). The Russian invasion of Ukraine have sent oil prices skyrocketing even further driven by oil trader speculation (See Watts this section). The boom returned during the economic recovery of 2021 – the same year the International Energy Agency (2021) proclaimed there should be *no* new oil investment if the world aims to reach net zero emissions by 2050 (See Eckhouse this section). Of course, international expert proclamations are one thing; the ongoing disasters of intensified fires, storms and heatwaves are quite another.

What can we make of this apparent disconnect of oil production gone wild alongside cascading climate disaster? One conclusion is that despite elite statements to the contrary, we cannot trust the hydrocarbon industry is acting in good faith. It seems clear that as long as fossil fuel investment is profitable, capital will continue investing in new projects (Christophers, 2022).

Thus, if we are to stave off the worst consequences of climate change, it appears we need a political force capable of *stopping* the oil industry. Most policy technocrats propose polite market interventions like carbon taxes. Not only are such measures politically unpopular (Mildenberger et al., 2022), they are of questionable efficacy (Green, 2021).

More radical proposals have called for *nationalization* of the industry (Buck, 2021; Gowan, 2018; Oil Change International and the Next System Project, 2020; Sweeney 2020). Such a proposal could be undertaken in two ways. Taxpayer money could be deployed to ‘buy out’ the existing industry. The other option is expropriation. While the latter sounds radical, I argue we have plenty of historical examples of *popular expropriation*. In all cases, expropriation was justified on the grounds of a history of injustice. Given the evidence of the oil industry's role in the climate crisis, it is not farfetched to say a political rationale for mass expropriation exists today. While a buyout might seem more politically feasible, it raises difficult questions of how to raise the fiscal capacity. One advocate for the buyout approach admitted, ‘[t]his would cost trillions and trillions of dollars’ (Spross, 2018). And, while we have seen trillions of fiscal spending as of late, skyrocketing inflation will no doubt bring out the austerity-mongers once again. If we could build the requisite popular energy, expropriation might be the more efficient option.

## Marx on expropriation

It is worth revisiting Marx's use of the concept of expropriation in *Capital*.^12^ There are three ways he uses it. First, in a minor comment on his notion of the concentration and centralization of capital he describes the ‘expropriation of capitalist by capitalist’ (Marx, [1867] 1976: 777).

Although this implies violence it is typically something accomplished through perfectly legal means. Second, he refers to the expropriation of the masses from any means of production.

Unlike the first definition, this ‘primitive accumulation’ entailed a form of expropriation based on coercion and violence (ibid: 874). We might refer to this as the ‘bad’ expropriation, and it seems the bulk of geographical and political ecological research focuses on this. Specifically, oil and gas industry scholars have paid special attention to the role dispossession of land and livelihoods near sites of oil extraction (e.g. Sawyer, 2004; Simpson, 2019).

Marx offered a third, and perhaps most important, understanding of expropriation. Near the end of *Capital* Volume 1 (Marx, [1867] 1976: 927–930), he suggests that along with the centralization of capital, ‘grows the revolt of the working class’ (ibid: 929). This mass movement then aims its sights on the shrunken power of capital. Marx declares, ‘The knell of capitalist private property sounds. The expropriators are expropriated’ (ibid).

This might be called the ‘good’ form of expropriation – that wielded by the working class to usher in revolutionary social control over production. Marx even sounds optimistic comparing this good form against primitive accumulation: ‘In the former case, it was…the expropriation of the mass of the people by a few usurpers; but in this case, we have the expropriation of a few usurpers by the mass of the people’ (ibid: 930). In our neoliberal era of capitalist realism (Fisher, 2009), few reference the ‘good’ expropriation. This might also be for good reason: the prospect of nationalized control of energy resources could certainly threaten alternative territorial sovereignties called for by indigenous movements (Radhuber and Radcliffe, 2022). That said, we can still learn from a rich history of energy and popular expropriation.

## A brief history of energy and popular expropriation

Throughout history movements have engaged in popular revolt targeting the wealth garnered from exploitation (see Lyall & Valdivia this section). First, while the ethical content diverges considerably, struggles for abolition were ultimately *energy struggles* over the enslavement of human muscle power (Hayes, 2014). While abolition often involved *compensating* slaveowners for their lost ‘property’, the emancipation of slaves in the U.S. did not do this for the bulk of the planter class.^13^ In other words, it was in effect a mass expropriation of the property embedded in 4 million slaves (Bourne, 2008). As W.E. B. DuBois (1935: 55) powerfully argued, this was achieved by the ‘general strike’ of the slaves themselves. Matt Karp (2019) posits antislavery as a form of ‘mass politics’ with the insurgent Republican Party mobilizing popular ideas of ‘free labor’ against the ‘slave power’ (which, abolitionists at the time recognized made up a mere 1% of society). This laid the conditions for what DuBois (1935: 434) articulated as an abandoned American experiment in ‘abolition democracy’ during Reconstruction.

There are also many examples of popular expropriation of the oil industry. While the first such example might be the Bolshevik nationalization of 1917, civil war, famine and economic collapse complicated matters. The communists were forced to partner with Standard Oil as soon as 1921 (Yergin, 1991: 240). An enduring example of popular expropriation occurred in Mexico in 1938. Culminating a long process that began with the start of the Mexican revolution decades before, Myrna I. Santiago (2006: 8) calls the nationalization a, ‘revolution “from below”’. Although the expropriation is often credited to the popular President Lázaro Cárdenas, Santiago (2006: 326) points out expropriation was, ‘a measure of last recourse for Cárdenas’ and, ‘it was the oil workers who ultimately led him to expropriation’ (ibid: 330). There was crushing level of strike activity leading up to the March 1938 decree (ibid: 333). Ultimately in March of 1938, it was a threat of a general strike that forced Cárdenas's hand. As such, ‘the oil workers rallied the entire country to their side and against the oil companies’ (ibid: 338). Zalik (2008: 182) rightly states, ‘It marked a key moment in the twentieth century between industrial capacity and state sovereignty in the Global South’.

The oil companies knew what Mexico meant. As Santiago (2006: 8) puts it, ‘preventing other Mexicos became a mantra among the transnational oil conglomerates’. Thirteen years later, after his election by parliament, Prime Minster Mohammad Mosaddegh nationalized the oil assets of the Anglo-Persian oil company (now British Petroleum). As Gregory Brew (2019: 44) explains, Mosaddegh ‘was one of the first postcolonial nationalist politicians’. At the core of Mosaddegh's political program was harnessing the popular *anger* at British oil profits draining Iranian wealth (ibid: 44). Mosaddegh's nationalization program was *not* a full blown expropriation since he offered compensation (Elm 1992: 285). But the takeover was concerning enough to lead the U.S. and British to organize the coup of 1953 and solidification of the Shah dictatorship until 1979.

The despotic rule of the Shah welcomed back international oil capital, and Iran's nationalization served as a lesson to other anti-colonial movements who dared to challenge the rule of capital. Yet, it did not stop other states from trying. As Christopher Dietrich (2017) shows, Arab states embarked on a multi-decade struggle to assert great sovereignty over oil. Of course, this is now a problem for climate activists, as these National Oil Companies (NOCs) have a vested interest in maintaining fossil capitalism.

## Conclusion

The experience of the climate crisis is often one of helplessness; of watching an unfolding disaster where it seems nothing can be done. But history shows popular movements can overcome the power of those who profit from energy systems. The epic mass struggles against slavery and colonialism utilized expropriation to usher in new social relations of production that changed history. Today, fossil capital continues to profit off the production of the very energy source making life uninhabitable on the planet. It is up to us to channel this injustice into a movement that insists we can and must stop them. The expropriators must be expropriated.

## References

[bibr78-0308518X221114879] BourneC (2008) “Slavery in the United States”. EH.Net Encyclopedia. http://eh.net/encyclopedia/slavery-in-the-united-states/

[bibr79-0308518X221114879] BlumenfeldJ (2022) Expropriation of the expropriators. Philosophy & Social Criticism. (Online First)

[bibr80-0308518X221114879] BrewG (2019) The collapse narrative: The United States, Mohammed Mossadegh, and the coup decision of 1953. Texas National Security Review2(4): 38–59.

[bibr81-0308518X221114879] BuckH (2021) Ending Fossil Fuels: Why Net Zero is Not Enough. London: Verso.

[bibr82-0308518X221114879] ChristophersB (2022) Fossilised capital: Price and profit in the energy transition. New Political Economy27(1): 146–159.

[bibr83-0308518X221114879] DietrichC (2017) Oil Revolution: Anticolonial Elites, Sovereign Rights, and the Economic Culture of Decolonization. New York: Cambridge University Press.

[bibr84-0308518X221114879] DuBoisWEB (1935) Black Reconstruction in America 1860–1880. New York: Free Press.

[bibr85-0308518X221114879] ElmM (1992) Oil, Power, and Principle: Iran’s Oil Nationalization and Its Aftermath. Syracuse: Syracuse University Press.

[bibr86-0308518X221114879] FisherM (2009) Capitalist Realism: Is There No Alternative?London: Zero Books.

[bibr87-0308518X221114879] GowanP (2018) A plan to nationalize fossil fuel companies. *People*’*s Policy Project*, 21 March.

[bibr88-0308518X221114879] GreenJ (2021) Does carbon pricing reduce emissions? A review of ex-post analyses. Environmental Research Letters16: 043004.

[bibr89-0308518X221114879] HayesC (2014) The new abolitionism. The Nation 22 April.

[bibr90-0308518X221114879] HunterT (2019) When slaveowners got reparations. The New York Times, 16 April.

[bibr91-0308518X221114879] International Energy Agency (2021) Net Zero by 2050: A Roadmap for the Global Energy Sector. Paris: IEA.

[bibr92-0308518X221114879] KarpM (2019) The mass politics of anti-slavery. Catalyst3(2): 131–178.

[bibr93-0308518X221114879] McCormickM (2022) Permian Basin: High oil price breathes new life into US shale. The Financial Times, 20 January.

[bibr94-0308518X221114879] MarxK (1867 1976) Capital Volume 1. London: Penguin.

[bibr95-0308518X221114879] MeredithS (2022) OPEC + agrees oil output hike from February as omicron Covid cases soar. CNBC.com, 4 January.

[bibr96-0308518X221114879] MildenbergerMLachapelleEHarrisonK, et al. (2022) Limited impacts of carbon tax rebate programmes on public support for carbon pricing. Nature Climate Change12: 141–147.

[bibr97-0308518X221114879] Oil Change International and the Next System Project (2020) “The Case for Public Ownership of the Fossil Fuel Industry” Discussion Paper available at: https://priceofoil.org/content/uploads/2020/04/Public-Ownership-Discussion-Paper_v5.pdf

[bibr98-0308518X221114879] RadhuberIRadcliffeS (2022) Contested sovereignties: Indigenous disputes over plurinational resource governance. Environment and Planning E: Nature and Space. (online first).

[bibr99-0308518X221114879] SantiagoM (2006) The Ecology of Oil: Environment, Labor, and the Mexican Revolution, 1900– 1938. New York: Cambridge University Press.

[bibr100-0308518X221114879] SawyerS (2004) Crude Chronicles: Indigenous Politics, Multinational Oil, and Neoliberalism in Ecuador. Durham, NC: Duke University Press.

[bibr101-0308518X221114879] SimpsonM (2019) Resource desiring machines: The production of settler colonial space, violence, and the making of a resource in the Athabasca tar sands. Political Geography74: 102044.

[bibr102-0308518X221114879] SprossJ (2018) Let’s buy out Big Oil. The Week, 17 December.

[bibr103-0308518X221114879] SweeneyS (2020) There may be no choice but to nationalize oil and gas—and renewables, too. New Labor Forum. Available online: https://newlaborforum.cuny.edu/2020/08/31/there-may-be-no-choice-but-to-nationalize-oil-and-gas-and-renewables-too/

[bibr104-0308518X221114879] YerginD (1991) The Prize: The Epic Quest for Oil, Money and Power. New York: Free Press.

[bibr105-0308518X221114879] ZalikA (2008) Oil sovereignties in the Mexican Gulf and Nigerian Niger Delta. In: OmejeK (ed) Extractive Economies and Conflicts in the Global South. London: Ashgate, pp. 181–198.
